# Activating *NTRK2* and *ALK* receptor tyrosine kinase fusions extend the molecular spectrum of pleomorphic xanthoastrocytomas of early childhood: a diagnostic overlap with infant-type hemispheric glioma

**DOI:** 10.1007/s00401-021-02396-y

**Published:** 2021-12-15

**Authors:** Calixto-Hope G. Lucas, Zied Abdullaev, Carol S. Bruggers, Kanish Mirchia, Nicholas S. Whipple, Mouied M. Alashari, Amy Lowichik, Samuel Cheshier, Joanna J. Phillips, Patrick Devine, David A. Solomon, Martha Quezado, Kenneth D. Aldape, Arie Perry

**Affiliations:** 1 Division of Neuropathology, Department of Pathology, University of California, 505 Parnassus Avenue, Room M551, San Francisco, CA 94143 USA; 2 Laboratory of Pathology, Center for Cancer Research, National Cancer Institute, Bethesda, MD USA; 3 Division of Pediatric Hematology/Oncology, Department of Pediatrics, University of Utah School of Medicine, Salt Lake City, UT USA; 4 Division of Pediatric Pathology, Department of Pathology, University of Utah School of Medicine, Salt Lake City, UT USA; 5 Division of Pediatric Neurosurgery, Department of Neurosurgery, University of Utah, Salt Lake City, UT USA; 6 Department of Neurological Surgery, University of California, San Francisco, CA USA; 7 Clinical Cancer Genomics Laboratory, University of California, San Francisco, CA USA

Pleomorphic xanthoastrocytoma (PXA) is a circumscribed glioma arising in the cerebral hemispheres of children and young adults. Recent molecular studies demonstrate an activating mitogen-activated protein (MAP) kinase mutation (most frequently *BRAF* p.V600E hotspot mutation) with co-occurring homozygous deletion of *CDKN2A* encoding the p16 cell cycle regulator protein in most PXA [11]. Less commonly, fusions involving *BRAF* and *RAF1* have previously been reported by our group [10]. Here, we present two young children with high-grade gliomas containing *CDKN2A/B* homozygous deletion and *NACC2*–*NTRK2* or *PPP1CB*–*ALK* fusion, along with DNA methylation signatures aligning to PXA. In both, the main differential diagnostic consideration was infant-type hemispheric glioma (IHG).

Patient #1: This 3-year-old boy presented with a 3-month history of progressive headaches, emesis, sound sensitivity and altered gait. Magnetic resonance imaging (MRI) demonstrated a 9.4 cm avidly enhancing, solid and cystic right temporal horn mass, extending into adjacent parenchyma and middle/posterior cranial fossa. He underwent gross total resection. Intraoperatively, the tumor appeared tan-yellow and vascular with extensive tentorial involvement. Histology revealed a mostly solid appearing spindled and epithelioid glial neoplasm arranged in fascicles, sheets, and papillae (Fig. 1a). Perivascular hyalinization was focally prominent and there were areas of palisading necrosis (Fig. 1b), microvascular proliferation, and up to 6 mitotic figures per 10 high-power fields. No definite eosinophilic granular bodies or Rosenthal fibers were seen. There were occasional multinucleate tumor cells. The tumor cells were extensively immunoreactive for GFAP (Fig. 1c), OLIG2, and CD34 (Fig. 1d), but negative for BRAF V600E mutant protein. There was no increased intercellular reticulin deposition. A neurofilament stain highlighted entrapped axons only at the tumor periphery and the p53 labeling index was 60%. A diagnosis of high-grade glioma was rendered with consideration of PXA, IHG, and astroblastoma. Additional molecular studies revealed *NACC2*–*NTRK2* fusion (Fig. 1e–f) and *CDKN2A/B* homozygous deletion (Supplementary Fig. 1 [Online Resource 1]). Post-operatively, the patient was treated with focal proton radiation to 55.8 Gy and concurrent temozolomide followed by 24 months of adjuvant daily oral larotrectinib (NTRK inhibitor) therapy. The patient is alive without evidence of disease 33 months following diagnosis.

**Fig. 1:**
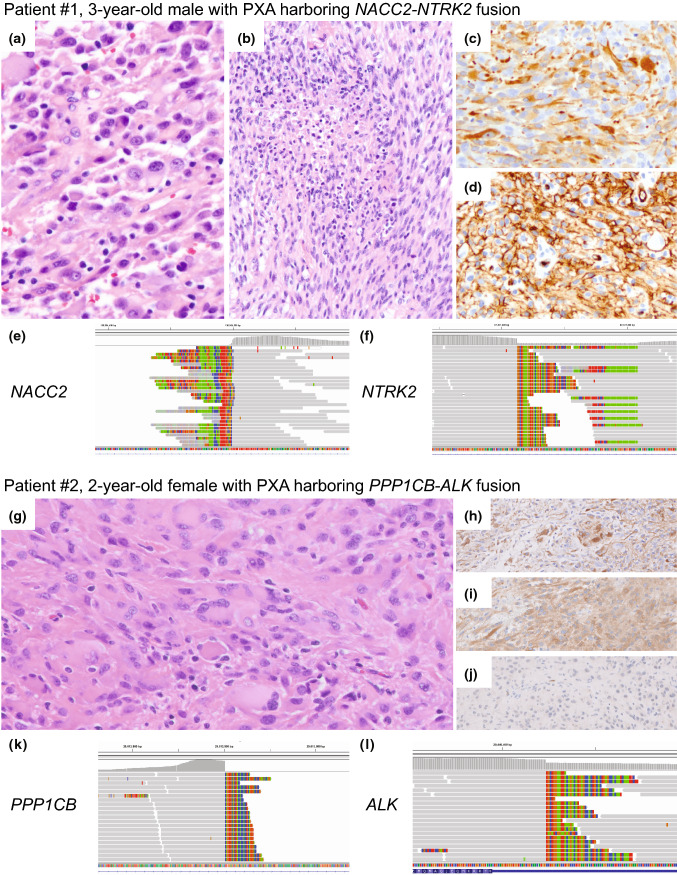
Histopathologic and molecular features of two pleomorphic xanthoastrocytomas harboring *NACC2*–*NTRK2* and *PPP1CB*–*ALK* receptor tyrosine kinase fusions. Patient #1, H&E sections revealed a high-grade glial neoplasm composed of pleomorphic tumor cells (**a**), along with areas of necrosis and an increased mitotic index (**b**). Immunohistochemical studies demonstrated GFAP immunoreactivity (**c**), supporting glial differentiation, as well as patchy immunoreactivity for CD34 (**d**). Next-generation sequencing results visualized in Integrative Genomics Viewer demonstrated fusion breakpoints in *NACC2* (**e**) and *NTRK2* (**f**). Patient #2, H&E sections revealed a solid glial neoplasm with frequent multinucleated cells (**g**) with GFAP-positivity (**h**), ALK-positivity (**i**), and lack of p16 protein expression by immunohistochemistry (**j**). Next-generation sequencing results demonstrated fusion breakpoints in *PPP1CB* (**k**) and *ALK* (**l**). Gray bar denotes sequence aligned to the reference human genome and multicolor bases denote mismatched base pairs that instead align with the fusion partner

Patient #2: This almost 3-year-old girl with longstanding mild gross motor and speech delays presented with a six-month history of episodic uncontrollable right arm shaking. MRI demonstrated a rounded 2.5 cm, partially cystic, enhancing left frontoparietal mass between the motor and sensory cortices. A gross total resection was achieved after two surgeries. The tumor was tan-red and well demarcated from adjacent brain parenchyma. Sections revealed a predominantly solid glial neoplasm with cells containing enlarged, irregular hyperchromatic nuclei, including scattered multinucleated forms (Fig. 1g). Areas of necrosis, microvascular proliferation, and up to 6 mitotic figures per 10 high-power fields were noted. No definite eosinophilic granular bodies or Rosenthal fibers were seen. Immunohistochemical stains demonstrated patchy positivity for GFAP (Fig. 1h), with extensive OLIG2, CD34, and ALK-positivity (Fig. 1i). The tumor cells were BRAF V600E negative and showed loss of p16 immunoreactivity (Fig. 1j). An initial diagnosis of high-grade glioma was rendered, with IHG (with a likely ALK alteration) and PXA being favored. Additional molecular studies revealed *PPP1CB*–*ALK* fusion (Fig. 1k–l) and *CDKN2A/B* homozygous deletion (Supplementary Fig. 1 [Online Resource 1]). Post-operatively, the patient (now age 3) was treated with focal proton radiation to 55.8 Gy and concurrent temozolomide followed by adjuvant daily oral lorlatinib (ALK inhibitor) therapy. The patient is alive without evidence of disease 6 months following diagnosis.

UCSF500 targeted next-generation sequencing (NGS) was performed as previously described [7]. Tumor #1 demonstrated a chromosome 2p inversion event resulting in a fusion involving the 5’ end of *NACC2* (exons 1–5 [transcript ID NM_144653]; Fig. 1e) and the 3’ end of *NTRK2* (exons 15–21 [transcript ID NM_006180], encoding the tyrosine kinase domain; Fig. 1f). Tumor #2 demonstrated a chromosome 9q inversion event resulting in a fusion involving the 5’ end of *PPP1CB* (exons 1–7 [transcript ID NM_206876]; Fig. 1k) and the 3’ end of *ALK* (exons 20–29 [transcript ID NM_004304], encoding the intracellular kinase domain; Fig. 1l). Both also demonstrated homozygous *CDKN2A/B* deletion (Supplementary Fig. 1 [Online Resource 1]). No other structural variants, focal chromosomal amplifications or deletions were noted. For both, DNA methylation profiling was performed as previously described [12] and demonstrated close proximity to a PXA reference cohort using tSNE dimensionality reduction analysis (Supplementary Fig. 2 [Online Resource 1]), with calibrated scores of > 0.99 matching to the PXA methylation class using both the 11b4 and 12.3 versions of the online DKFZ random forest classification algorithm (molecularneuropathology.org). The integrated diagnosis for both was PXA, CNS WHO grade 3.

Given the young patient ages, the diagnosis of IHG was considered initially. This new entity in the 5^th^ edition of the CNS WHO [2] presents in early childhood (< 1 year of age) with enrichment for receptor tyrosine kinase (RTK) fusions involving *NTRK1/2/3*, *ROS1*, *ALK* or *MET* [4, 5, 8]. The same *PPP1CB*–*ALK* fusion identified here has been described in two IHGs [1, 5, 9]. However, both patients were less than 1 year of age, and neither harbored *CDKN2A/B* homozygous deletion. *NTRK* family fusions have also been reported in rare pilocytic astrocytomas [6]. Of note, a pediatric PXA was reported to harbor an *ETV6*–*NTRK3* fusion along with *CDKN2A* homozygous deletion [13]. The same *NACC2*–*NTRK2* fusion with *CKDN2A* homozygous deletion identified here has recently been described in a pediatric cerebellar high-grade glioma [3]. Ancillary DNA methylation studies were not performed in those cases.

Given the non-canonical molecular profiles in our two cases, the integrated diagnosis was heavily weighted on the DNA methylation profiling results. These cases extend the molecular spectrum of anaplastic PXA to include tumors harboring *CDKN2A/B* homozygous deletion with accompanying RTK fusions rather than MAP kinase alterations. A small subset of PXA do not harbor *BRAF* or *RAF1* alterations, and these may contain RTK fusions that are only inconsistently detected using conventional NGS methods. Additional molecular studies could be informative to more accurately categorize these challenging pediatric lesions which may otherwise be classified as IHG or diffuse pediatric-type high-grade glioma, H3-wildtype, and IDH-wildtype according to contemporary criteria [2]. Although both of these patients demonstrate no recurrent disease following gross total resection and treatment with focal proton radiation followed by adjuvant NTRK and ALK inhibitor targeted therapy, this report is limited by short follow-up data. Patients with recurrent disease may benefit from personalized molecularly based therapeutic strategies and enrollment in precision medicine clinical trials. These cases underscore the importance of an integrated histologic and molecular approach for the accurate diagnosis and optimal treatment of pediatric glioma patients.

In summary, the fusions identified here illustrate that a subset of PXA can harbor RTK fusions rather than MAP kinase alterations, which extends the molecular spectrum of this tumor type beyond what is currently recognized [2]. In addition, we highlight the diagnostic challenge in differentiating RTK-fused PXA and IHG given our novel findings that RTK fusion can be seen in both tumor types.

## Supplementary Information

Below is the link to the electronic supplementary material.

Supplementary file1 (PDF 910 kb)

## Data Availability

Raw sequencing and DNA methylation data files are available from the authors upon request.
