# Supplementary material for: Activating NTRK2 and ALK receptor tyrosine kinase fusions extend the molecular spectrum of pleomorphic xanthoastrocytomas of early childhood: a diagnostic overlap with infant-type hemispheric glioma
Source: Acta Neuropathol. 2021 Dec 15;143(2):283–6. doi: 10.1007/s00401-021-02396-y (PMC8742815; doi:10.1007/s00401-021-02396-y)
Supplement: Supplementary file 1 — Supplementary file1 (PDF 910 kb) [file 401_2021_2396_MOESM1_ESM.pdf]

Patient #1, 3-year-old male with PXA harboring *NACC2-NTRK2* fusion

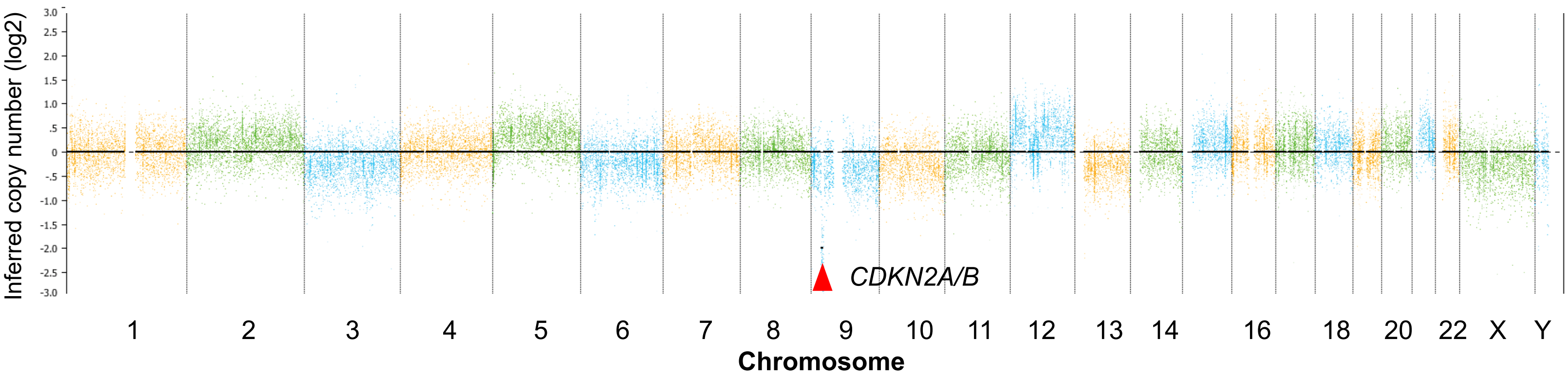

Patient #2, 2-year-old female with PXA harboring *PPP1CB-ALK* fusion

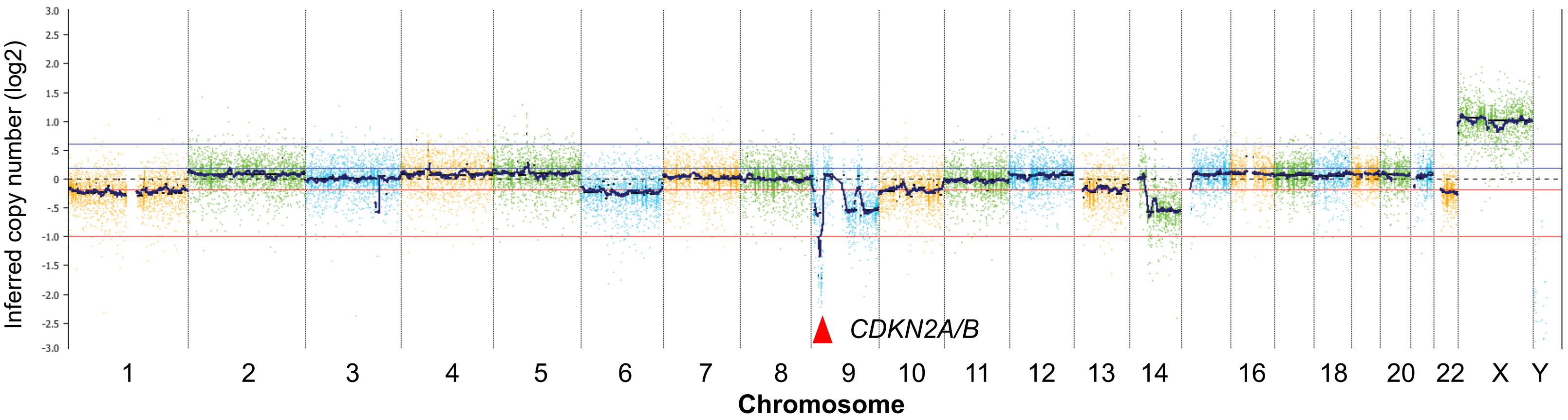

Supplementary Figure 1. Chromosomal copy number plot for a PXA harboring an *NACC2-NTRK2* gene fusion and focal homozygous deletion of *CDKN2A/B* (above). Large-scale chromosomal copy number changes include gains of interstitial 2q, 5, 12, 15q, 16, and 21q, as well as losses of 3, 9, and 13q. Chromosomal copy number plot for a PXA harboring a *PPP1CB-ALK* fusion and focal homozygous deletion of *CDKN2A/B* (below). Lare-scale chromosomal copy number changes include losses of 1, 6, 9, 10, 13q, 14q, and 22q.

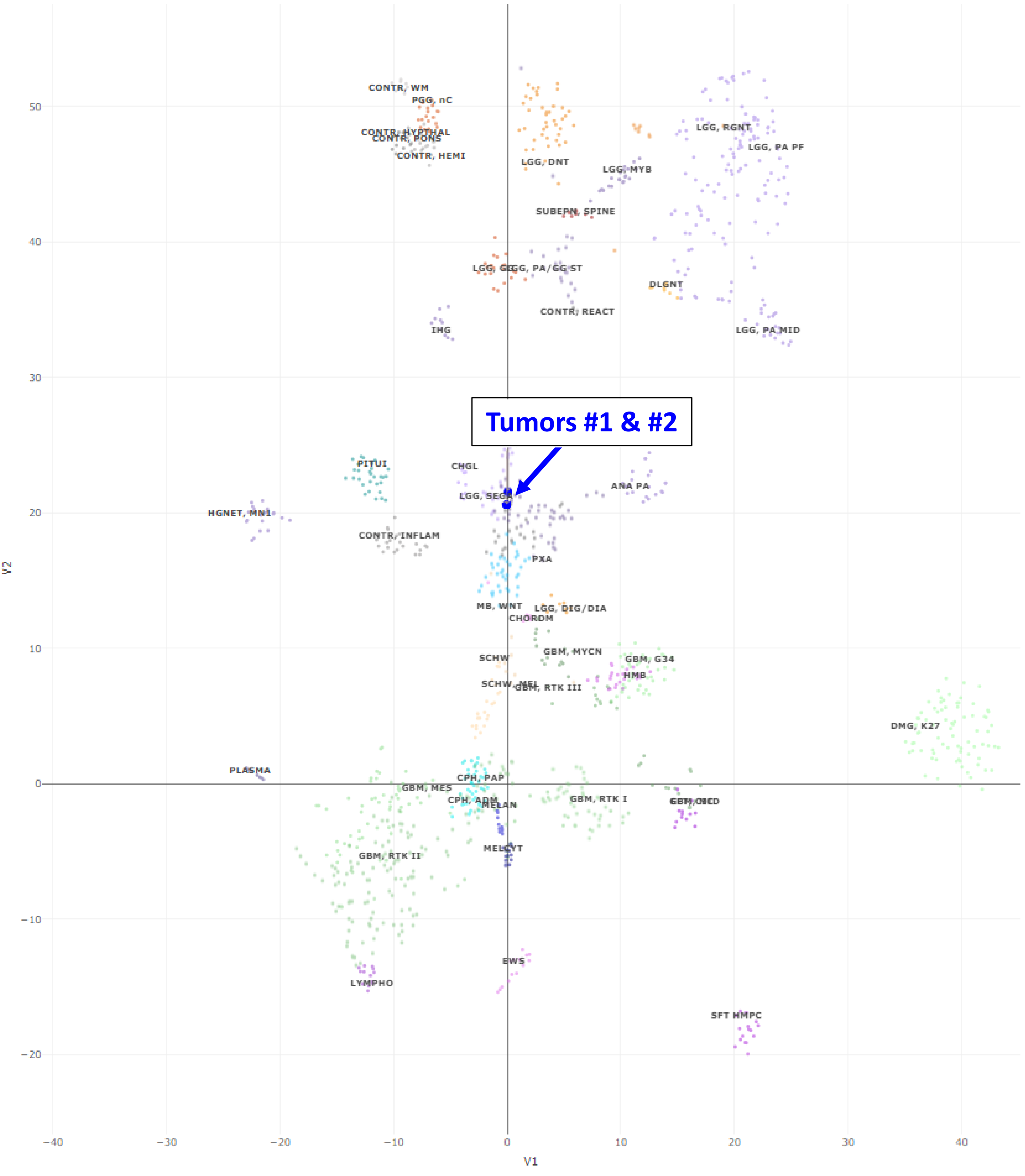

Supplementary Figure 2. tSNE plot of genome-wide DNA methylation profiles from tumors #1 and #2 alongside a cohort of reference CNS tumor entities demonstrates epigenetic signatures most similar to the methylation class “PXA; pleomorphic xanthoastrocytoma” and distant from “IHG; infant-type hemispheric glioma” and other glioma types.
